# Patient‐centered communication tool for older patients with acute myeloid leukemia, their caregivers, and oncologists: A single‐arm pilot study

**DOI:** 10.1002/cam4.5547

**Published:** 2022-12-19

**Authors:** Marissa LoCastro, Chandrika Sanapala, Ying Wang, Marielle Jensen‐Battaglia, Marsha Wittink, Sally Norton, Heidi D. Klepin, Daniel R. Richardson, Jason H. Mendler, Jane Liesveld, Eric Huselton, Kristen O'Dwyer, Ashley‐Marie Cortes, Chrystina Rodriguez, William Dale, Kah Poh Loh

**Affiliations:** ^1^ School of Medicine and Dentistry University of Rochester Rochester New York USA; ^2^ Burrell College of Osteopathic Medicine Las Cruces New Mexico United States; ^3^ Department of Public Health Sciences University of Rochester Medical Center Rochester New York USA; ^4^ Department of Psychiatry University of Rochester School of Medicine and Dentistry Rochester New York USA; ^5^ School of Nursing University of Rochester Medical Center Rochester New York USA; ^6^ Section on Hematology and Oncology Wake Forest Baptist Comprehensive Cancer Center, Medical Center Blvd Winston‐Salem North Carolina USA; ^7^ Division of Hematology, Department of Medicine, Lineberger Comprehensive Cancer Center University of North Carolina Chapel Hill North Carolina USA; ^8^ Division of Hematology/Oncology, Department of Medicine James P. Wilmot Cancer Institute, University of Rochester Medical Center Rochester New York USA; ^9^ Department of Supportive Care City of Hope National Medical Center Duarte California USA

**Keywords:** acute myeloid leukemia, communication tool, decision aid, shared decision making

## Abstract

**Background:**

In a single‐arm pilot study, we assessed the feasibility and usefulness of an innovative patient‐centered communication tool (UR‐GOAL tool) that addresses aging‐related vulnerabilities, patient values, and prognostic awareness for use in treatment decision making between older adults with newly diagnosed acute myeloid leukemia (AML), their caregivers, and oncologists.

**Methods:**

Primary feasibility metric was retention rate; >50% was considered feasible. We collected recruitment rate, usefulness, and outcomes including AML knowledge (range 0–14) and perceived efficacy in communicating with oncologists (range 5–25). Due to the pilot nature and small sample size, hypothesis testing was performed at *α* = 0.10.

**Results:**

We included 15 patients (mean age 76 years, range 64–88), 12 caregivers, and 5 oncologists; enrollment and retention rates for patients were 84% and 73%, respectively. Patients agreed that the UR‐GOAL tool helped them understand their AML diagnosis and treatment options, communicate with their oncologist, and make more informed decisions. From baseline to post‐intervention, patients and caregivers scored numerically higher on AML knowledge (patients: +0.6, *p* = 0.22; caregivers: +1.1, *p* = 0.05) and perceived greater efficacy in communicating with their oncologists (patients: +1.5, *p* = 0.22; caregivers: +1.2, *p* = 0.06).

**Conclusion:**

We demonstrated that it is feasible to incorporate the UR‐GOAL tool into treatment decision making for older patients with AML, their caregivers, and oncologists.

## Graphical abstract

We demonstrated that it is feasible to incorporate a patient‐centered communication tool (UR‐GOAL) into treatment decision making. For patients and caregivers, AML knowledge scores and perceived efficacy in communicating with the oncologist increased at post‐intervention.

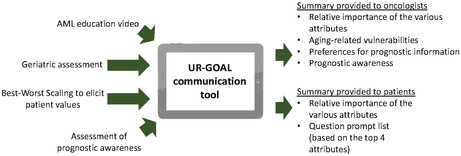

## 1 INTRODUCTION

Acute myeloid leukemia (AML) is the most common type of acute leukemia; 70% of new cases occur in adults aged ≥60 years.^1^ Recently, more effective and tolerable treatment options have become available for older adults with AML who are “unfit” for standard induction chemotherapy.^2^ Nonetheless, aging‐related vulnerabilities (i.e., physical impairments) are prevalent among older adults, which may limit the potential benefit of treatment.^3^, ^4^ Older patients with comorbidities and poor performance status have been underrepresented in therapeutic clinical trials^5^; thus the ideal treatment approach for these patients is unclear and strongly informed by patient preferences.^6^ In the absence of comparative data, it is challenging to identify older patients who may benefit from low‐intensity treatments, or who may experience excessive treatment toxicities.^7^ Practice patterns vary widely, leading to overtreatment, undertreatment, and poor outcomes in this population.^7^, ^8^ Incorporation of aging‐related vulnerabilities into AML treatment selection may improve outcomes by informing treatment decision making.^9^

Shared decision making (SDM), an approach where clinicians and patients share the best available evidence when making decisions, and where patients are supported to consider options, is essential to achieve patient‐centered care.^10^, ^11^ SDM is associated with greater patient‐rated quality of care, satisfaction with physician communication, and patient‐reported outcomes.^6^ To achieve high quality SDM, patients must be knowledgeable about their disease, treatment options, and prognosis, and their values should be incorporated into treatment decisions.^12^ Achieving SDM can be challenging given the sudden onset of AML, and treatment decisions must be made quickly.^13^ In a previous qualitative study of older adults with AML, we found that many patients believed they were not well informed about their disease and its treatment options, which is a barrier to achieving high‐quality SDM.^14^

Accurate prognostic awareness is an important component of SDM. In a multicenter prospective observational study, over half of the patients with hematologic malignancies overestimated their chance of cure compared to their oncologists' estimates.^15^ Patients with poor prognostic awareness, who generally overestimate their prognostic survival, are more likely to opt for aggressive chemotherapy but experience no additional benefit in survival.^16^ A standardized process of eliciting prognostic awareness may help tailor communication about prognosis to support SDM.

Preference elicitation techniques, such as best‐worst scaling (BWS), can be used to assess the relative importance patients place on various aspects or attributes of care.^17^ These techniques consist of choice tasks involving treatment attributes such as daily activities, quality of life, location of treatment, and survival. Preference elicitation techniques have been used with patients with AML to identify the strength of preferences for different outcomes.^18^, ^19^ These methods may improve SDM by clarifying patient values and allowing oncologists to align treatments with what matters most to individual patients.^20^

We previously developed and adapted a patient‐centered communication tool [University of Rochester‐Geriatric Oncology Assessment for acute myeloid Leukemia (UR‐GOAL)] in older adults with AML.^21^ The UR‐GOAL tool addresses aging‐related vulnerabilities, patient values, and prognostic awareness in older adults with AML, with the goal of promoting SDM. In this study, we assessed the feasibility and usefulness of the UR‐GOAL tool for use in treatment decision making between older adults with newly diagnosed AML, their caregivers, and oncologists.

## 2 METHODS

### 2.1 Study Design, Setting, and Participants

In this single‐arm pilot study, we recruited older patients with AML, their caregivers, and oncologists from the University of Rochester Medical Center Wilmot Cancer Institute (WCI) and its affiliated oncology practices in upstate New York. Eligible patients were (1) aged ≥60 years; (2) newly diagnosed with AML; (3) English‐speaking; and (4) capable of providing informed consent. Eligible caregivers (if available) were (1) aged ≥21 years and (2) a family member, partner, friend, or caregiver with whom the patient discusses health‐related matters. Patients could enroll without their caregivers, but caregivers could enroll only if their respective patient consented to the study. The inclusion criterion for oncologists was practicing oncologist with at least one of their patients enrolled in the study. Participants were recruited between October 2021 and March 2022. Eligibility was confirmed by the treating physician and the principal investigator (KPL). The study was approved by the University of Rochester Research Subjects Review Board.

### 2.2 Study procedures

Once eligibility was confirmed, study personnel approached patients and caregivers face‐to‐face or via telephone. Following consent, patients, and their caregivers completed demographic and clinical measures. Cancer and treatment information for patients was obtained from the electronic medical record. Patients completed the UR‐GOAL intervention (with their caregivers if available) prior to the clinical encounters that involved treatment discussion with their oncologists. At post‐intervention (within 4 weeks after a treatment decision was made), patients and caregivers completed measures as well as a 60‐min semi‐structured interview virtually or in a private space in the hospital. Interviews focused on experience with and feedback on the UR‐GOAL tool. Interviews were transcribed verbatim and uploaded to MAXQDA software (VERBI Software GmbH) for analyses.

### 2.3 Intervention description

**FIGURE 1:**
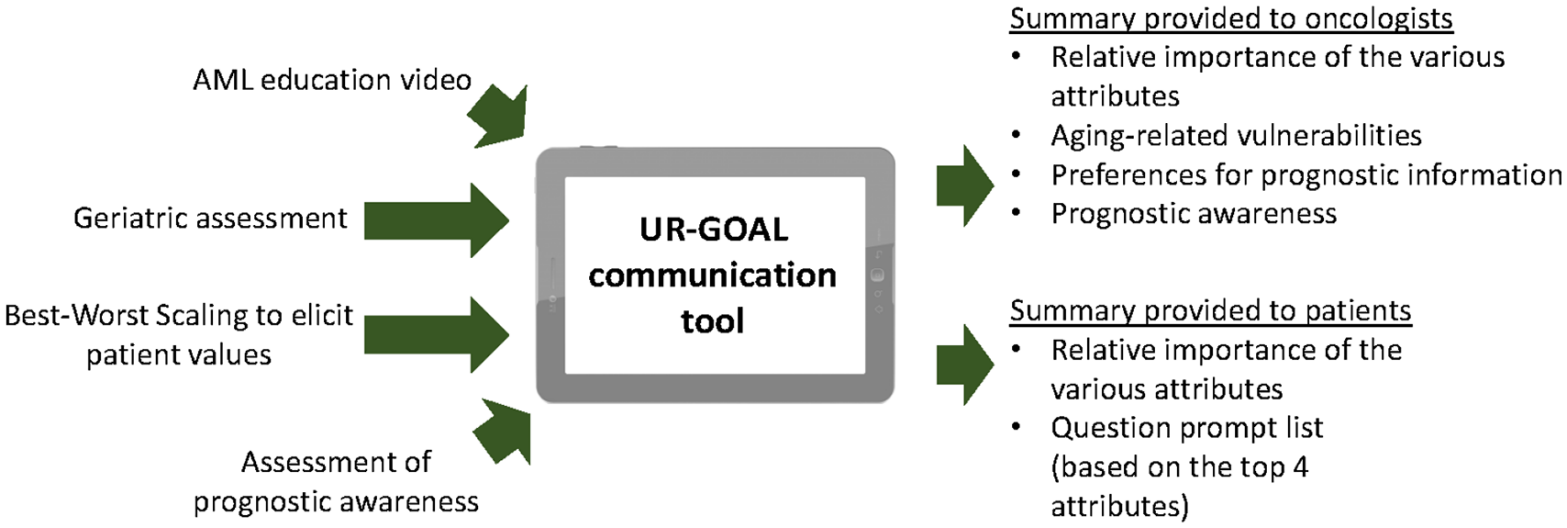
Components of the UR‐GOAL communication tool.

The UR‐GOAL communication tool has previously been described and has four components (Figure 1): (1) An AML educational video, (2) Geriatric assessment (GA) to assess aging‐related vulnerabilities, (3) Preference elicitation using Best‐Worse Scaling (BWS), and (4) Prognostic awareness.^21^ Separate summary reports are generated for patients and oncologists at completion.

#### 2.3.1 AML educational video

The 5‐min animated video addresses diagnosis, incidence, symptoms, risk factors, goals of treatment, treatment approaches, and population‐based prognosis. The content was informed by stakeholders in our prior studies.^14^, ^22^

#### 2.3.2 Geriatric assessment

To assess aging‐related vulnerabilities, patients completed the self‐report component of a GA that included measures of physical function, nutritional status, social support, and psychological health. Physical function was assessed using the Katz Activities of Daily Living (ADL), Older Americans Resources and Services (OARS) Instrumental ADL, and number of falls in the previous 6 months.^23^, ^24^ Nutritional status was measured using self‐reported weight loss in the last 6 months and body mass index.^23^ Patients were asked about their living situation and their main support. Psychological health was measured using the Geriatric Depression Scale‐15 (GDS‐15).^25^

Objective tests of physical function [Short Physical Performance Battery (SPPB)] and cognitive function (Mini‐Cog) were performed by study personnel, either in person or over a tele‐video platform (virtual SPPB was used for the latter).^26^ The SPPB consists of gait speed, chair stand, and balance tests, with scores ranging from 0 to 12; scores <10 are considered impaired.^27^ The Mini‐Cog includes a three‐word recall task and clock drawing, with scores ranging from 0–5; <4 is considered impaired.^28^ Results from tests were entered into the UR‐GOAL tool.

#### 2.3.3 Preference elicitation

Preference elicitation using the BWS technique was performed using Sawtooth Software (Sawtooth Software, Inc.,). The BWS consists of 10 choice tasks, with four attributes (out of eight potential attributes) per task (Figure S1). The eight attributes were daily activities, quality of life, location of treatment, survival, thinking/memory, response to treatment, time to remission, and risk of death from treatment. Patients were presented with four different attributes at a time and asked to select the most and least important attribute when choosing a treatment.

#### 2.3.4 Assessment of prognostic awareness and preferences for receiving prognostic information

Prognostic awareness (i.e., patients' estimates of their percent chance of cure and overall life expectancy) and preferences for prognostic information (i.e., whether a conversation about prognosis would be helpful to them) were assessed. These questions were adapted from previous studies that included more than 1000 older patients with cancer.^15^, ^29^

#### 2.3.5 Summary report

After completion of the UR‐GOAL tool, a summary report was provided to the patient (caregiver can also review the same report) and the oncologist. Both reports contained attributes from BWS grouped into the four most important and four least important attributes. The patient report included question prompts based on the most important attributes. The oncologist report contained a summary of the GA and the patient's prognostic awareness.

### 2.4 Measures

#### 2.4.1 Feasibility and usefulness

The primary feasibility metric was retention rate. We considered the study feasible if >50% of patients who consented to the study ultimately completed the study intervention and post‐intervention assessment. We captured recruitment rate (percentage of patients who were approached and agreed to enroll) and usefulness. Usefulness was assessed using the Preparation for Decision Making Scale (mean score range from 1 to 5, and higher scores indicate greater usefulness) and a self‐developed survey.^30^

#### 2.4.2 Other measures

Measures obtained at baseline and post‐intervention for patients and caregivers included AML knowledge, efficacy in communication, psychological health, and prognostic awareness. Measures obtained at post‐intervention only included patient‐centered communication and shared decision making.

#### 2.4.3 Patient measures

##### Baseline and post‐intervention

AML knowledge was assessed by a 14‐item questionnaire developed by the investigators. Scores range from 0–14 (higher scores are better). Efficacy in communication was measured using the 5‐item Perceived Efficacy in Patient‐Physician Interactions (PEPPI) questionnaire. Scores range from 5–25 (higher scores are better).^31^ Psychological health was assessed using the Generalized Anxiety Disorder‐7 (GAD‐7) and GDS‐15. GAD‐7 scores range from 0 to 21 (higher scores indicate greater anxiety).^32^ GDS‐15 scores range from 0–15 (higher scores indicate greater depression). Prognostic awareness included patients' self‐perceived disease curability and life expectancy.

##### Post‐intervention only

SDM was measured using the Shared Decision‐Making (SDM‐Q‐9) and 3‐item CollaboRATE. We transformed scores to a scale of 0 to 100.^33^, ^34^ We utilized the Patient‐Centered Communication in Cancer Care (PCC‐Ca‐36) to assess patient‐centered communication (range 1–5; higher is better).^35^ Decisional Conflict Scale was used to assess patients' uncertainty in decision making; scores were converted to a 0–100 scale (higher scores indicate greater decisional conflict).^36^

#### 2.4.4 Measures for caregivers

Similar measures were used for caregivers [i.e., Perceived Efficacy in Caregiver‐Physician Interactions (PECPI)], AML knowledge, GAD‐7, prognostic awareness, adapted 6‐item Patient‐Centered Communication in Cancer Care, CollaboRATE).^37^, ^38^ Depressive symptoms were assessed using the 2‐item Patient Health Questionnaire (PHQ‐2). Scores range from zero (no depression mood at all) to six (depression mood nearly every day).

#### 2.4.5 Measures for oncologists

At post‐intervention, oncologists completed a self‐developed four‐item usefulness survey regarding the UR‐GOAL tool and summary report.

### 2.5 Analyses

A priori, we chose 15 as our sample size which is considered sufficient based on recommendations from previous feasibility and usability studies.^39^, ^40^ Descriptive statistics were used to summarize sample characteristics. We used paired *t*‐tests for measures with normally distributed changes and Wilcoxon signed‐rank tests for measures with non‐normally distributed changes to conduct the comparisons between baseline and post‐intervention. Because of the pilot nature of the study and small sample size, hypothesis testing was performed at *α* = 0.10 (2‐tailed). All quantitative analyses were conducted using SAS statistical software, version 9.4 (SAS Institute Inc.,).

Content analysis was used to analyze the post‐intervention interviews.^41^ Two independent coders analyzed all transcripts using the MAXQDA 2018 software. We created an initial coding schema which was revised in subsequent transcripts until thematic saturation was met. Discrepancies were resolved through consensus.

## 3 RESULTS

### 3.1 Demographics

We approached 19 patients and 16 consented (recruitment rate: 84%). Of the three patients who did not consent, two did not feel well, and one was overwhelmed. One patient withdrew between consent and baseline assessment due to being overwhelmed, resulting in 15 patients. Twelve caregivers and five oncologists were enrolled.

Mean age of patients was 76 years (SD 8, range 64–88); 47% (7/15) were male, 93% (14/15) were white and non‐Hispanic, and 67% (10/15) were married. At diagnosis, 60% (9/15) had European LeukemiaNet high‐risk AML.^42^ Forty percent (6/15) received intensive treatment, 47% (7/15) received lower intensity treatment, and 13% (2/15) received best supportive care (one of these patients chose lower intensity treatment but received best supportive care because of health decline). Demographics and clinical characteristics are in Table 1. Seven patients completed the UR‐GOAL tool while inpatient, and eight patients completed it while outpatient; two out of eight outpatients had previously seen a community oncologist. Prevalence of aging‐related vulnerabilities are in Table S1.

**TABLE 1:** Patients' demographics and clinical characteristics.

Variable		*N* = 15
Age in years, mean (SD, range)		76 (8.0, 64–88)
Gender, *n* (%)	Male	7 (46.7)
	Female	8 (53.3)
Race, *n* (%)	Black or African American	1 (6.7)
	White	14 (93.3)
Ethnicity, *n* (%)	Not Hispanic or Latino	14 (93.3)
	Unknown/Not Reported	1 (6.7)
Education, *n* (%)	High school or below	5 (33.4)
	Training after high school or at least some college/university	3 (20.0)
	College/University graduate	5 (33.3)
	Postgraduate level	2 (13.3)
Marital status, *n* (%)	Married	10 (66.7)
	Single or widowed	5 (33.4)
Employment status, *n* (%)	Employed	3 (20.0)
	Retired or homemaker	12 (80.0)
Living arrangement, *n* (%)	Independent living (more than 1 story)	6 (40.0)
	Independent living (1 story)	8 (53.3)
	Assisted Living	1 (6.7)
Household members, *n* (%)	Live alone	2 (13.3)
	Spouse/partner	10 (66.7)
	Parent(s), children, or other relative	3 (20.0)
ECOG performance status (KPS), *n* (%)	0 (90–100)	3 (20.0)
	1 (70–80)	6 (40.0)
	2 (50–60)	5 (33.3)
	3 (30–40)	1 (6.7)
Upfront AML treatment, *n* (%)	Intensive treatment	6 (40.0)
	Lower intensity treatment	7 (46.7)
	Best supportive care	2 (13.3)
*AML risk* group *n* (%)	Intermediate	6 (40.0)
	High	9 (60.0)
Antecedent hematologic malignancies, *n* (%)	Yes	3 (20.0)
	No	12 (80.0)

Abbreviations: AML, acute myeloid leukemia; ECOG, Eastern Cooperative Oncology Group; KPS, Karnofsky Performance Status.

Mean age of the 12 caregivers was 69 years (SD 13, range 47–86); half were male and all were white. Over half (67%, 8/12) were the patient's partner, and 33% (4/12) were children of the patient (Table 2). Demographics for oncologists are not reported due to small sample size.

**TABLE 2:** Caregivers' demographics

Variable		*N* = 12
Age in years, mean (SD, range)^a^		69 (12.7, 47–86)
Gender, *n* (%)	Male	6 (50.0)
	Female	6 (50.0)
Race, *n* (%)	White	12 (100.0)
Ethnicity, *n* (%)	Not Hispanic or Latino	11 (91.7)
	Unknown/Not Reported	1 (8.3)
Education, *n* (%)	Training after high school or at least some college/university	4 (33.3)
	College/University graduate	4 (33.3)
	Postgraduate level	4 (33.3)
Marital status, *n* (%)	Married	10 (83.3)
	Long term, committed significant other	2 (16.7)
Employment status, *n* (%)	Employed	5 (41.7)
	Retired or homemaker	7 (58.3)
Relationship to the patient, *n* (%)	Partner	8 (66.7)
	Child/Children or other relative	4 (33.3)

^a^ One caregiver declined to provide age.

### 3.2 Feasibility and usefulness

Of the 15 patients consented, 11 completed both the study intervention and assessments (retention rate: 73%). Of the four patients who did not complete both, three patients completed the study intervention but not the post‐intervention assessments (two transitioned to hospice and one withdrew due to not feeling well). One patient withdrew while completing the study intervention because of distress. Table 3 shows the distribution of responses on the patient usefulness questionnaire. All patients strongly agreed or agreed that the UR‐GOAL tool helped them understand their health (100.0%). Majority strongly agreed or agreed that the UR‐GOAL tool helped them understand AML and its treatment options (90.9%), identify questions to ask their doctor (81.8%), understand their personal values (91.0%), talk to their doctor (90.9%), and make better decisions (90.9%) (Table 3). Mean usefulness scores on the Preparation for Decision Making Scale were 3.8 (SD 0.7) and 3.5 (1.2) for patients and caregivers, respectively. Oncologists found the UR‐GOAL tool to be useful and helped to understand the patient's fitness level (100.0%), discuss acute myeloid leukemia and its treatment options (100.0%), and provide patient‐centered care (100.0%) (Table 4).

**TABLE 3:** Patient perceived usefulness of the UR‐GOAL tool (*N* = 11).

	Strongly agree	Agree	Neutral	Disagree	Strongly disagree
The UR‐GOAL tool helps you to better understand your health.	3 (27.3%)	8 (72.7%)	0 (0.0%)	0 (0.0%)	0 (0.0%)
The UR‐GOAL tool helps you to better understand about acute myeloid leukemia and its treatment options.	6 (54.5%)	4 (36.4%)	1 (9.1%)	0 (0.0%)	0 (0.0%)
The UR‐GOAL tool helps you to identify questions you want to ask your doctor.	3 (27.3%)	6 (54.5%)	2 (18.2%)	0 (0.0%)	0 (0.0%)
The UR‐GOAL tool helps you to better understand your personal values (personal values are the things that are important to you which guide your decision. Example of personal values include maintaining your independence or living longer)	5 (45.5%)	5 (45.5%)	0 (0.0%)	1 (9.1%)	0 (0.0%)
The UR‐GOAL tool prepares you to talk to your doctor about what matters most to you.	4 (36.4%)	6 (54.5%)	1 (9.1%)	0 (0.0%)	0 (0.0%)
The UR‐GOAL tool makes you feel more informed about acute myeloid leukemia and its treatment options.	6 (54.5%)	3 (27.3%)	2 (18.2%)	0 (0.0%)	0 (0.0%)
The UR‐GOAL tool helps you to make a better decision.	4 (36.4%)	6 (54.5%)	1 (9.1%)	0 (0.0%)	0 (0.0%)
The UR‐GOAL tool helps to meet your needs.	4 (36.4%)	6 (54.5%)	1 (9.1%)	0 (0.0%)	0 (0.0%)
The UR‐GOAL tool helps you to be more certain about the treatment.	4 (36.4%)	6 (54.5%)	1 (9.1%)	0 (0.0%)	0 (0.0%)

**TABLE 4:** Oncologist perceived usefulness of the UR‐GOAL tool (*N* = 3).

	Strongly agree	Agree	Neutral	Disagree	Strongly disagree
The UR‐GOAL tool/summary helps me to better understand the patient's fitness level.	2 (66.7%)	1 (33.3%)	0 (0.0%)	0 (0.0%)	0 (0.0%)
The UR‐GOAL tool/summary helps me to discuss acute myeloid leukemia and its treatment options.	1 (33.3%)	2 (66.7%)	0 (0.0%)	0 (0.0%)	0 (0.0%)
The UR‐GOAL tool/summary helps me to choose a treatment for the patient.	1 (33.3%)	0 (0.0%)	2 (66.7%)	0 (0.0%)	0 (0.0%)
The UR‐GOAL tool/summary helps me to achieve patient centered care.	1 (33.3%)	2 (66.7%)	0 (0.0%)	0 (0.0%)	0 (0.0%)

### 3.3 Measures at baseline and/or post‐intervention

**FIGURE 2:**
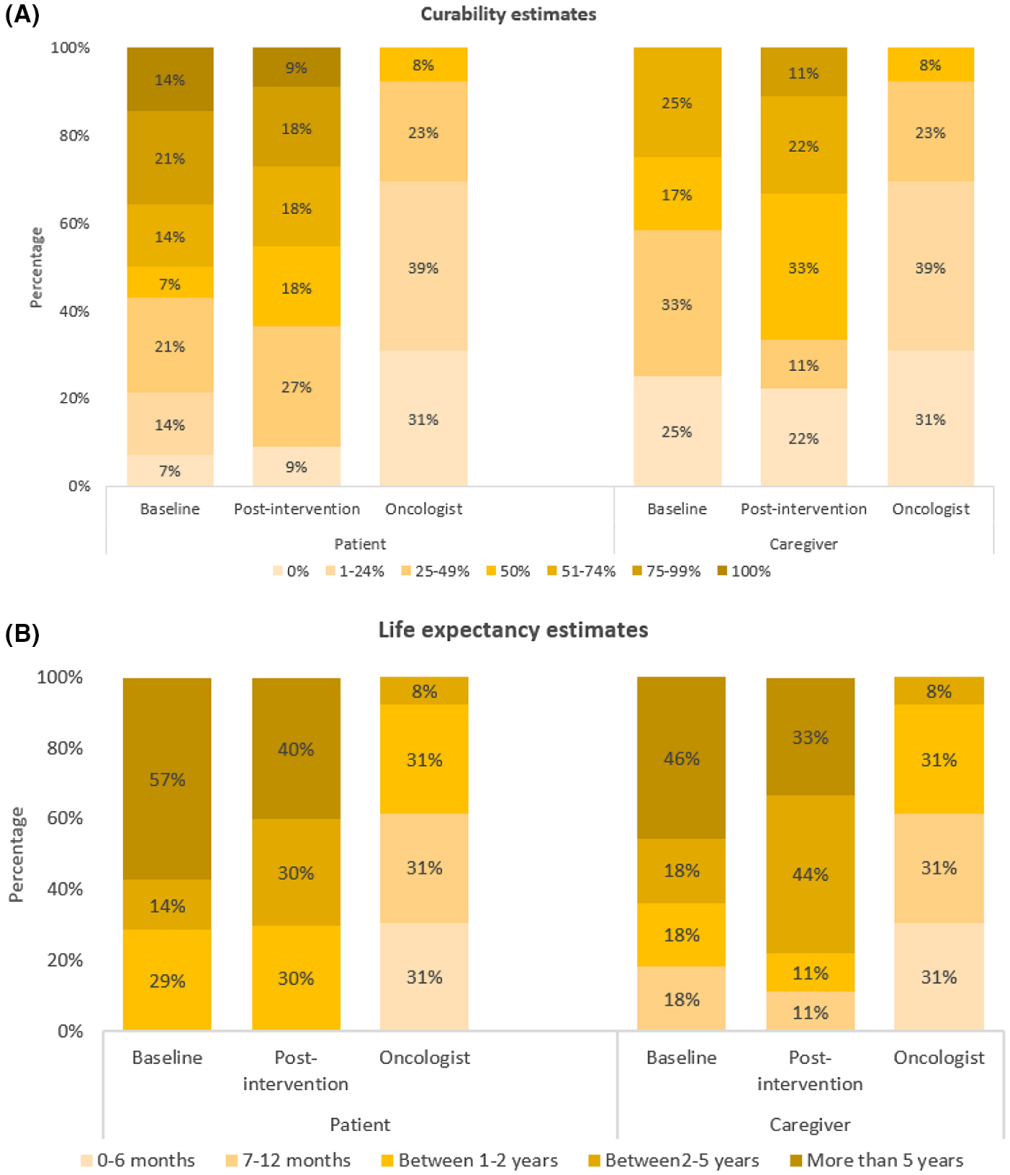
Prognostic awareness among older patients, caregivers, and oncologists. (A) Curability estimates; (B) Life expectancy estimates.

Mean AML knowledge scores among patients and caregivers were 6.0 (SD 1.1) and 5.7 (1.3), respectively, which increased to 6.5 (SD 1.5; *p* = 0.22) and 6.9 (SD 1.1; *p* = 0.05) at post‐intervention (Table 5). Patients and caregivers also reported higher perceived efficacy in communicating with their oncologist [mean change of +1.5 (SD 3.0) among patients (*p* = 0.22) and mean change of +1.2 (SD 1.6) among caregivers (*p* = 0.06)]. GAD‐7 scores decreased numerically in both patients and caregivers [mean change of −1.6 (SD 5.4) among patients (*p* = 0.34) and mean change of −2.2 (SD 4.8) among caregivers (*p* = 0.20)]. Other measures available at post‐intervention only are shown in Table 5. Patients and caregivers were more optimistic than the oncologists in perceptions of curability and life expectancy (Figure 2).

**TABLE 5:** Baseline and/or post‐intervention measures.

	Baseline			Post‐intervention			Change mean from baseline to post‐intervention			*p*‐value^a^
	Mean (SD)	Median (IQR)	*N*	Mean (SD)	Median (IQR)	*N*	Mean (SD)	Median (IQR)	*N*	
Patients (*N* = 15)										
AML knowledge^b^	6.0 (1.1)	6.0 (2.0)	13	6.5 (1.5)	7.0 (2.0)	10	0.6 (1.4)	0.0 (2.0)	10	0.22
Geriatric Depression Scale‐15^c^	2.9 (2.9)	2.0 (3.0)	15	2.6 (2.5)	2.0 (4.0)	11	0.2 (2.1)	0.0 (2.0)	11	0.78
Generalized Anxiety Disorder‐7^c^	7.1 (6.3)	7.0 (14.0)	15	5.1 (4.7)	3.0 (10.0)	11	−1.6 (5.4)	−1.0 (4.0)	11	0.34
Perceived efficacy in patient‐physician interactions^b^	20.4 (4.0)	20.5 (3.0)	14	21.2 (4.4)	23.0 (5.0)	11	1.5 (3.0)	0.0 (5.0)	10	0.22
Patient‐centered communication in cancer care‐36^b^				4.4 (0.5)	4.6 (0.8)	11				
Shared decision‐making‐Q9^b^				89.4 (14.4)	95.6 (13.3)	9				
CollaboRATE measure^b^				97.5 (7.4)	100.0 (0.0)	9				
Decisional conflict scale^c^				9.1 (9.6)	6.3 (15.6)	11				
Caregivers (*N* = 12)										
AML knowledge^b^	5.7 (1.3)	6.0 (1.0)	11	6.9 (1.1)	7.0 (2.0)	9	1.1 (1.5)	1.0 (2.0)	9	0.05
Perceived efficacy in caregiver‐physician interactions^b^	21.3 (2.6)	22.0 (3.0)	12	22.6 (1.9)	23.0 (2.0)	9	1.2 (1.6)	1.0 (3.0)	9	0.06
Patient health questionnaire‐2^c^	0.6 (0.9)	0.0 (1.5)	12	3.4 (1.0)	4.0 (1.0)	9	2.8 (1.1)	3.0 (2.0)	9	<0.01
Generalized Anxiety Disorder‐7^c^	6.8 (5.8)	8.0 (10.0)	11	4.8 (2.5)	5.0 (3.0)	9	−2.2 (4.8)	−2.0 (7.0)	9	0.20
Patient‐centered communication in cancer care‐6^b^				4.1 (0.8)	4.3 (1.0)	8				
CollaboRATE Measure^b^				93.5 (8.1)	96.3 (11.1)	8				

^a^ Wilcoxon Signed Rank test was used for Perceived efficacy in Patient‐physician interactions and paired t‐tests were used for all the other measures.

^b^ Higher score is better.

^c^ Higher score is worse.

### 3.4 Qualitative feedback from post‐intervention interviews

#### 3.4.1 Patients and Caregivers

Participants felt that the educational video was easily understandable, but two participants found it challenging to hear about the prognostic information (i.e., “less than 20% of patients with AML survive two years or more after their AML diagnosis”). They felt reassured when the range of prognoses based on treatment were discussed with their oncologist. An opportunity to re‐watch the educational video was suggested (of note, patients in this study did not re‐watch the video, but suggested it for future participants).

Three participants (two patients and one caregiver) felt that the BWS questions in the UR‐GOAL communication tool were repetitive, and one patient found it challenging to select the best and worst attributes.

Patient 5 (Female, 72‐years‐old): “I think it's too long…I know you were rephrasing it in a way that makes sure the answer stayed, but the rephrasing could – I just thought it was too long”.

Patients found the summary report helpful, and one patient reported using the summary report as a reference during visits with their oncologist. Patients and caregivers appreciated the question prompts provided.

Patient 15 (Male, 64‐years‐old): “Yes. In particular, the list of questions on there. We had that thing right in front of us and we used those”.

#### 3.4.2 Oncologists

Oncologists felt that the UR‐GOAL tool was most useful if used early in the treatment discussion (i.e., ideally before the first visit), but that it could also be used to confirm decisions that had already been made.

Oncologist 2: “She did really well in the geriatric assessment, and she perceived herself as being very strong, which made me feel better having given her pretty strong chemo to try and get back in remission”.

The tool provided oncologists with insight into the patients' understanding (or lack of understanding) regarding their diagnosis and prognosis, as well as their aging‐related vulnerabilities. Oncologists did not perceive the tool to be burdensome and the summary report was easy to interpret. Oncologists suggested adding specific details on aging‐related vulnerabilities (e.g., instead of reporting screening positive for depression on GDS‐15, include scores with clarification about the severity of depression and if intervention is warranted).

## 4 DISCUSSION

We found that it was feasible to recruit older patients with AML, their caregivers, and oncologists to a single‐arm pilot study testing a patient‐centered communication tool at diagnosis. Patients reported that the tool helped them to better understand their diagnosis and treatment options, and prepare for decision making. Oncologists found the UR‐GOAL tool to be useful and provided them with valuable insight into the patient's understanding of their diagnosis. The UR‐GOAL tool may improve patient and caregiver knowledge of AML and their perceived efficacy in communicating with their physician.

Due to the sudden diagnosis of AML and the high prevalence of aging‐related vulnerabilities, treatment decision making is challenging for older patients.^3^, ^4^ A communication tool may facilitate treatment conversations between older patients, their caregivers, and oncologists, but may not be feasible during the high stress period at diagnosis. In this study, we demonstrated that it is feasible to incorporate a tool to help promote treatment discussions. Four previous studies have investigated tools to facilitate decision making in AML; two included only patients with AML^43^, ^44^ and two studies included a mixed cancer population including AML.^45^, ^46^ Interventions included trained facilitators providing treatment decision support, a web‐based tool and nurse‐delivered telephone support, actual patient videos sharing experiences, and AML educational videos. In the study by Hildenbrand et al., 100% of participants with AML completed all study components, supporting the feasibility of using a decision support tool in this setting.^43^ Our tool is unique because it specifically focuses on older adults with AML and collects information about aging‐related vulnerabilities, patient values, and prognostic awareness, in addition to providing an AML education video.

Our findings suggest that the UR‐GOAL tool may improve patient and caregiver knowledge of AML as well as patient and caregiver perceived efficacy in interacting with their oncologist, consistent with other studies of decision aids for patients with cancer. A previous systematic review of 87 studies found that use of decision aids for patients with cancer is associated with increased patient knowledge.^47^ In a single‐arm pilot study of patients with AML (*n* = 20 patients, mean age = 62.4 years), use of a decision aid (animated videos on AML and treatment) improved knowledge scores from pre‐ to post‐intervention.^43^ Another randomized controlled trial of a web‐based communication tool for patients with lymphoma found that patients' perceived efficacy in communicating with oncologists in the intervention arm was improved compared to the control arm (1.97 points on a range of 10–50; *p* = 0.02).^48^

We demonstrated that the UR‐GOAL tool may promote SDM, improve patient‐centered communication, and reduce decisional conflict. At post‐intervention, patients reported a mean score of 89.4 (SD 14.4) on SDM‐Q‐9 (a measure of SDM). While we do not have a comparison arm, previous studies exploring SDM for treatment decisions have reported a range of 11–100, with a median score of 84 in a study of patients with hematologic malignancies.^49^ Mean scores on PCC‐Ca (a measure of patient‐centered communication) were 4.4 and 4.1 (out of five) for patients and caregivers, respectively, at post‐intervention. In a cross‐sectional study of patients with ovarian cancer, the mean score on PCC‐Ca was 4.09.^50^ At post intervention, mean decisional conflict score for our patients was 9.1 (SD 9.6). A previous study testing a decision aid for AML found that decisional conflict scores decreased from baseline (28.5, SD 24.1) to post‐intervention (22.1, SD 17.3; *p* = 0.02).^43^ Our preliminary scores on these measures were comparable or higher than the scores from patients with cancer in previously published literature. Of note, while prognostic awareness may have improved from baseline to post‐intervention, patients and caregivers remained more optimistic than the oncologists in perceptions of curability and life expectancy. This is not surprising given prognostic awareness is generally challenging to improve, and interventions to address prognostic awareness need to target multiple levels including the patient, caregiver, healthcare professional, societal, and systemic.^51^ A future randomized controlled trial will evaluate the effect of the UR‐GOAL tool on SDM, communication, and other outcomes.

Our study has several strengths. First, we included older patients with AML, their caregivers (who are often involved in the decision‐making process), and oncologists. Previous studies on decision aids have not routinely included caregivers. Second, we utilized both quantitative and qualitative methods to better understand the UR‐GOAL tool. Third, we used multiple reliable measures to generate a comprehensive assessment of the tool.

This study has limitations. First, this was a single‐arm pilot study without a comparison arm. While we were able to assess some outcomes at baseline and post‐intervention (e.g., AML knowledge), changes in other outcomes such as SDM and decisional conflict cannot be measured at baseline since the decision‐making process had not yet occurred. Second, our study sample was small, so appropriate caution is needed when extrapolating these findings to other settings. Third, while we attempted to enroll patients prior to the first encounter addressing treatment decisions, some patients may have met with a community oncologist and initiated these discussions. Fourth, the study participants in this group were predominantly white, non‐Hispanic, and English speaking. The results may not be generalizable to patients of other races and ethnicities.

In conclusion, we demonstrated that it is feasible to incorporate a patient‐centered communication tool in clinical practice for treatment decision making for older patients with AML, their caregivers, and oncologists. The UR‐GOAL tool may improve patient and caregiver knowledge of AML and perceived efficacy in interacting with the oncologist. Findings have allowed us to adapt our study procedures and the tool, which has informed an ongoing pilot randomized control trial (NCT05335369) to evaluate the preliminary efficacy of the UR‐GOAL tool on SDM and communication. Innovative interventions, such as the UR‐GOAL tool, which address challenges specific to older adults with AML are critical to improve health outcomes for this vulnerable population.

## AUTHOR CONTRIBUTIONS

**Marissa LoCastro:** Formal analysis (equal); writing – original draft (lead); writing – review and editing (lead). **Chandrika Sanapala:** Data curation (equal); formal analysis (supporting); project administration (equal); writing – review and editing (supporting). **Ying Wang:** Data curation (equal); formal analysis (equal); writing – review and editing (supporting). **Marielle Jensen‐Battaglia:** Data curation (supporting); formal analysis (equal); writing – review and editing (supporting). **Marsha Wittink:** Conceptualization (supporting); investigation (supporting); methodology (supporting); writing – review and editing (supporting). **Sally A Norton:** Conceptualization (supporting); investigation (supporting); methodology (supporting); writing – review and editing (supporting). **Heidi Klepin:** Conceptualization (supporting); investigation (supporting); methodology (supporting); writing – review and editing (supporting). **Daniel R Richardson:** Writing – review and editing (supporting). **Jason H Mendler:** Writing – review and editing (supporting). **Jane L. Liesveld:** Writing – review and editing (supporting). **Eric Huselton:** Writing – review and editing (supporting). **Kristen O'Dwyer:** Writing – review and editing (supporting). **Ashley‐Marie Cortes:** Formal analysis (supporting); writing – review and editing (supporting). **Chrystina Rodriguez:** Formal analysis (supporting); writing – review and editing (supporting). **William Dale:** Writing – review and editing (supporting). **Kah Poh Loh:** Conceptualization (lead); data curation (equal); formal analysis (equal); funding acquisition (lead); investigation (lead); methodology (lead); resources (lead); writing – original draft (equal); writing – review and editing (equal).

## FUNDING INFORMATION

This work was supported by the National Cancer Institute at the National Institutes of Health (UG1CA189961; K99CA237744l and R00CA237744 to KPL), the National Institute of Aging at the National Institutes of Health (R33AG059206 to HDK), Conquer Cancer American Society of Clinical Oncology, and Walther Cancer Foundation Career Development Award (to KPL), and the Wilmot Research Fellowship Award (to KPL). The content of this report is solely the responsibility of the authors and does not necessarily represent the official views of the National Institutes of Health.

## CONFLICT OF INTEREST

Dr. Loh has served as a consultant to Pfizer and Seattle Genetics and has received honoraria from Pfizer. All other authors have no relevant competing interests to report.

## Supporting information


Appendix S1.

Figure S1.

Table S1.

## Data Availability

For original data, please contact kahpoh_loh@urmc.rochester.edu.
