# Supplementary material for: Patient‐centered communication tool for older patients with acute myeloid leukemia, their caregivers, and oncologists: A single‐arm pilot study
Source: Cancer Med. 2022 Dec 19;12(7):8581–93. doi: 10.1002/cam4.5547 (PMC10134384; doi:10.1002/cam4.5547)
Supplement: Supplementary file 1 — Appendix S1. [file CAM4-12-8581-s001.pdf]

Patient-centered communication tool for older patients with acute myeloid leukemia, their caregivers, and oncologists: A single-arm pilot study

Appendices:

Patient AML Knowledge…………………………………...…………………...……. Page 2

Patient Disease Understanding……………………………………………………..…. Page 3

Caregiver AML knowledge…………………………………………………….…... Page 4

Caregiver Disease Understanding…………………………………………………….. Page 5

**UR-GOAL**<br>AML Knowledge	

○ Baseline ○ Post-intervention

Instructions: Please circle "Yes" or "No" to the best of your abilities.

Acute myeloid leukemia is a type of blood cancer.	**Yes / No**
The likelihood of getting acute myeloid leukemia increases with age.	**Yes / No**
Acute myeloid leukemia does not generally affect my blood cells (white blood cells, red blood cells, and platelets).	**Yes / No**
I can catch acute myeloid leukemia from someone else.	**Yes / No**
Previous use of chemotherapy and/or radiation may increase the risk of getting AML.	**Yes / No**
Treatment of acute myeloid leukemia is generally divided into three categories: intensive, lower-intensity, and best supportive care.	**Yes / No**
Intensive treatment for acute myeloid leukemia requires me to be hospitalized for 4-5 weeks.	**Yes / No**
Lower-intensity treatments are generally given in the outpatient setting (i.e., in the form of injection/infusion or pills).	**Yes / No**
Best supportive care means that I would not be receiving blood transfusion.	**Yes / No**
Regardless of the treatment I choose, I may be hospitalized for complications (e.g., infections) related to my acute myeloid leukemia and treatment.	**Yes / No**
In general, the only cure for acute myeloid leukemia is hematopoietic stem cell transplantation.	**Yes / No**
Hematopoietic stem cell transplantation is well-tolerated in older adults with acute myeloid leukemia.	**Yes / No**
Remission means that I am cured from my acute myeloid leukemia.	**Yes / No**
Most older adults with acute myeloid leukemia are cured with treatment (i.e., the AML will not return).	**Yes / No**

UR-GOAL
Understanding of Disease - Patient

○ Baseline ○ Post-Intervention

1. What do you believe are the chances your cancer will go away and never come back <u>with treatment</u>?

☐ 100% ☐ 75 - 99% ☐ 51% - 74% ☐ 50%

☐ 25% - 49% ☐ 1% - 24% ☐ 0%

2. Considering your health, and your underlying medical conditions, what do you estimate <u>your overall</u> life expectancy to be?

☐ 0 to 6 months ☐ 7 to 12 months ☐ Between 1 to 2 years

☐ Between 2 to 5 years ☐ More than 5 years

UR-GOAL<br>AML Knowledge - Caregiver	

○ Baseline ○ Post-intervention

Instructions: Please circle "Yes" or "No" to the best of your abilities.

Acute myeloid leukemia is a type of blood cancer.	**Yes / No**
The likelihood of getting acute myeloid leukemia increases with age.	**Yes / No**
Acute myeloid leukemia does not generally affect the blood cells (white blood cells, red blood cells, and platelets).	**Yes / No**
The patient can catch acute myeloid leukemia from someone else.	**Yes / No**
Previous use of chemotherapy and/or radiation may increase the risk of getting AML.	**Yes / No**
Treatment of acute myeloid leukemia is generally divided into three categories: intensive, lower-intensity, and best supportive care.	**Yes / No**
Intensive treatment for acute myeloid leukemia requires me to be hospitalized for 4-5 weeks.	**Yes / No**
Lower-intensity treatments are generally given in the outpatient setting (i.e., in the form of injection/infusion or pills).	**Yes / No**
Best supportive care means that the patient would not be receiving blood transfusion.	**Yes / No**
Regardless of the treatment chosen by the patient, he/she may be hospitalized for complications (e.g., infections) related to my acute myeloid leukemia and treatment.	**Yes / No**
In general, the only cure for acute myeloid leukemia is hematopoietic stem cell transplantation.	**Yes / No**
Hematopoietic stem cell transplantation is well-tolerated in older adults with acute myeloid leukemia.	**Yes / No**
Remission means that the patient is cured from his/her acute myeloid leukemia.	**Yes / No**
Most older adults with acute myeloid leukemia are cured with treatment (i.e., the AML will not return).	**Yes / No**

**UR-GOAL**<br>Disease Understanding - Caregiver	

○ Baseline ○ Post-intervention

Instructions: The following are questions about what you believe about the patient's illness. There are no right or wrong answers. They ask about the patient's quality of life and how long you think the patient might live. We understand that it might be difficult to answer some of these questions and we appreciate you making your best guess.

1. What do you believe are the changes that the patient's cancer will go away and never come back with treatment?

☐ 100% ☐ 75 - 99% ☐ 51% - 74% ☐ 50%

☐ 25% - 49% ☐ 1% - 24% ☐ 0%

2. Considering the patient's health, and underlying medical conditions, what would <u>you</u> estimate the patient's overall life expectancy to be?

☐ 0 to 6 months ☐ 7 to 12 months ☐ Between 1 to 2 years

☐ Between 2 to 5 years ☐ More than 5 years
