# Supplementary material for: Patient‐centered communication tool for older patients with acute myeloid leukemia, their caregivers, and oncologists: A single‐arm pilot study
Source: Cancer Med. 2022 Dec 19;12(7):8581–93. doi: 10.1002/cam4.5547 (PMC10134384; doi:10.1002/cam4.5547)
Supplement: Supplementary file 2 — Figure S1. [file CAM4-12-8581-s003.doc]

Supplemental Figure 1
Please consider how important the priorities below are to you when choosing a cancer treatment. Considering only these 4 priorities, choose ONE that is MOST IMPORTANT and choose ONE that is LEAST IMPORTANT?

Most Important
Least Important


0	Location of treatment	0
Whether a treatment requires a one-month stay at the hospital versus receiving it in the hospital and going home on the same day


0	0
Chance of surviving treatment-related complications
How likely it is that I will survive from complications resulting from treatment


Time to remission

How long it will take before my cancer goes into remission G


0	0
Survival
How likely it is that I will be alive one year or more after treatment
