# Supplementary material for: Patient‐centered communication tool for older patients with acute myeloid leukemia, their caregivers, and oncologists: A single‐arm pilot study
Source: Cancer Med. 2022 Dec 19;12(7):8581–93. doi: 10.1002/cam4.5547 (PMC10134384; doi:10.1002/cam4.5547)
Supplement: Supplementary file 3 — Table S1. [file CAM4-12-8581-s002.doc]

Supplemental Table 1: Geriatric assessment

Geriatric assessment domain and measure	Description of domain and measure	Mean (SD) or
Median
(IQR)	Definitions of impairment	N=15, n with impairment (%)	
Physical function			At least one impairment in SPPB, ADL,
IADL, and fall	13 (86.7)	
SPPBa	Three tasks that include balance, gait speed, chair stands); evaluates lower extremity function, scores range from 0-12;
lower score is worse	7.5
(SD 3.5)	 9	6 (54.5)	
Virtual SPPBb	Assesses patients' perceived ability to perform the three tasks on SPPB, scores range from
0-12; lower score is
worse	4.3
(SD 4.3)	 9	3 (75.0)	
ADL	Katz ADL; assesses independence in six self-care activities (e.g., bathing, ambulating), scores range from 0-6;
lower score is worse	6.0
(IQR 1.0)	<6	5 (33.3)	
IADL	OARS IADL;
assesses independence in seven self-care activities that are more complex (e.g., preparing meals, managing finances), scores range from
0-14; lower score is worse	13.0 (IQR
3.0)	<14	10 (66.7)	

Fall	Fall history over the
past year		 1	3 (20.0)	
Nutritional status	BMI and self- reported weight loss in the prior 6 months	BMI: 26.6
(SD 5.2)	A BMI <21 or
>10% weight loss in the prior 6 months	3 (20.0)	
		Weight loss in kg: -
1.8 (IQR
2.7)			
Comorbidities	OARS Comorbidity Scale; patients report the presence or absence of 15 comorbidities and how the
comorbidities affect them	3.4
(SD 1.8)	≥1 comorbidities that affect them a “great deal”, or ≥3 comorbidities	11 (73.3)	
Psychological health	Geriatric Depression scale; a 15-item valid and reliable screening tool for depression in older adults,
higher score is worse	2.0
(IQR 3.0)	 5	3 (20.0)	
Social supportc	MOS Social Support survey: patients indicate support in four questions (if they had someone to help if they were confined to bed, take them to the doctor if needed, prepare their meals if they were unable to do it, and help with daily chores if they were sick), scores range from
4-20; higher score is better	18.0 (IQR
4.0)	Patients selected some of the time, a little of the time, or none of the time for any of the four questions	4 (28.6)	
Medications	Number of
scheduled and as needed medications	4.0
(IQR 7.0)	≥5 scheduled
and as needed medications	7 (46.7)	

	excluding chemotherapy		excluding chemotherapy		
Cognitionc	Mini-Cog; a 3-item screening tool for cognitive
impairment in older adults	0.0
(IQR 0.0)	Recall 0 word
or recall 1-2 words but abnormal clock
drawing test	2 (14.3)	
a11 participants b4 participants c14 participants

Abbreviations: ADL, Activities of Daily Living; BMI, body mass index; IADL, Instrumental Activities of Daily Living; MOCA, Montreal Cognitive Assessment; MOS, Medical Outcomes Survey; OARS, Older American Resources and Services; SPPB, Short Physical Performance Battery
